# Fire risk assessment in fire hazardous zones of gasoline stations

**DOI:** 10.1002/1348-9585.12137

**Published:** 2020-07-27

**Authors:** Sunisa Chaiklieng, Thawatchai Dacherngkhao, Pornnapa Suggaravetsiri, Vichai Pruktharathikul

**Affiliations:** ^1^ Department of Environmental Health, Occupational Health and Safety Faculty of Public Health Khon Kaen University Khon Kaen Thailand; ^2^ Doctor of Public Health Program Candidate Faculty of Public Health Khon Kaen University Khon Kaen Thailand; ^3^ Department of Epidemiology and Biostatistics Faculty of Public Health Khon Kaen University Khon Kaen Thailand

**Keywords:** fire, occupational health, risk factor, safety, vapor recovery systems

## Abstract

**Objective:**

This cross‐sectional study aimed to assess fire risk in fire hazardous zones (FHZ) at the dispenser areas of gasoline stations.

**Methods:**

There were 47 stations chosen for fire risk assessment and two zones around the dispenser area of each station were assessed. The assessments were carried out by applying the matrix (3 × 4) of fire risk assessment by multipliers of opportunities level of hazard occurrence and the subsequent severity of the incident.

**Results:**

Across the 47 gasoline stations studied, there was an average of 23 ± 12 nozzles and none had vapor recovery systems (VRS) on dispenser nozzles. The average daily amount of gasoline sold was 3382 L. Each gasoline station had an average of 10 ± 5 workers/station; they all worked within a 1.5 meter radius of the dispenser (FHZ‐I); and they used cellphones >4 hours/day. The average level of flammable gas was in the range 1.3%‐7.4% LEL‐UEL (6.2% ± 5.2%). The fire risk was found to be an intolerable risk in FHZ‐I at 40 stations (85.1%) and FHZ‐II at 10 stations (21.3%). A total of 18 stations were ranked as having a substantial risk, whereas 19 stations also had a moderate risk in FHZ‐II; those levels correlated with the station locations and the quantity of fuel sold.

**Conclusion:**

It suggests that high risk must be controlled by using preventive and safety actions: eliminating fire ignition sources, such as by cellphone prohibition, and inspection of electrostatic discharges, engineering control with grounding when refuelling, signposting of hazardous zones, and VRS installation on dispenser nozzles.

## 1 INTRODUCTION

There are 40.19 million fuel‐powered vehicles registered as cars in Thailand,^1^ consuming the equivalent of about 24 000 tons of crude oil in the transportation sector. The number of gasoline stations has risen by an average of 4‐5 percent a year to 28 753 stations in 2019.^2^ This situation could cause potential health risks due to an increase of fuel vapor emissions and poor quality of ambient air at gasoline stations. Ambient air at gasoline stations contains volatile organic compounds (VOCs) from fuel vapors and combustion, including those of benzene, toluene, ethyl benzene, and xylene (BTEX).^3^ Previous studies showed that the annual average contribution to VOCs from fuel evaporation was 21.5% ± 0.99%.^4^ There have been studies on the impact of benzene vapor on gasoline station workers’ health in Thailand. For example, with regard to the health risk from air benzene exposure, it was shown that 15.3% of gasoline station workers were at a higher than acceptable level of risk,^5^ even though the benzene concentration was not higher than the standard occupational exposure limit (OEL‐TWA; <0.1 ppm) set by NIOSH.^6^ According to tt‐muconic acid (tt‐MA) biomarker monitoring, 36.7% of workers were at a higher than acceptable risk level,^7^ although it had been found that only 12% of those workers had a detected concentration of tt‐MA higher than the biological exposure index (>500 microgram per gram of creatinine), recommended by ACGIH.^8^

This situation not only has impacts on health, but it might also cause a fire risk at gasoline stations. The cause of fires has been related to chemical reactions initiated by energy ignition (smoking, mobile sparks, static electricity, heat transfer of surface material, etc), oxygen concentration, and fuel vapor (flammable gas vapor).^9^ Fire is a type of catastrophic disaster, causing damage to lives and properties, and air pollution around the world. Another study reported interesting statistics on unsafe behavior that could lead to accidents at gasoline stations. In Taiwan, the level of occupational safety control at gasoline stations was lower than 80%.^10^ In Nigeria, 65% of gasoline stations and their workers had no acknowledged training in safety and 45% of the gasoline stations did not follow the established requirements of legislation.^11^ Another report highlighted 87 cases (20%) of fire caused by an electric spark ignition source, 112 cases (25.75%) caused by storage tanks, 222 cases (51.03%) caused by loading and unloading operations, and 331 cases (76.09%) caused by fuel mixtures from a total of 435 fire explosion accidents at oil depots in China between 1951 and 2013.^12^

In Thailand, the safety control of gasoline stations is managed by the Ministry of Energy, which determines precautionary regulations for safe distance and usage of standard electronic devices, based on the risks of the hazardous area.^13^ The legislation controls electrical apparatus standards in hazardous zones at gasoline stations. Hazard levels are used to divide the gasoline station area into two zones (zone I and zone II). The legislation and study mentioned above provide a measure of fire prevention at gasoline stations. However, there have not yet been any studies on fire risk assessment (FRA) in fire hazardous zones (FHZ) at gasoline stations in Thailand, which is the main objective of this study.

## 2 MATERIALS AND METHODS

### 2.1 Sample size

This study was conducted at 47 gasoline stations. The representative sample size of the gasoline stations was calculated using Cochran^14^ under the known number of a total of 64 stations in Khon Kaen province, Thailand, which met the inclusion criteria of the study.^2^ There were two inclusion criteria for the gasoline stations; the station had to be (1) located in an area along either side of, or within 5 km of Mittraparp Road in Khon Kaen province, Thailand; and (2) have more than eight dispenser nozzles. This study was approved by Khon Kaen University's Ethics Committee for Human Research (No. HE612102).

### 2.2 Data collection

Data records were collected through a questionnaire and a survey on safety and occupational management of the gasoline stations, the fire risk behavior of gasoline station workers and measurement of fire risk factors. Fire risk factors, including levels of flammable gases, heat ignition sources, electrostatic discharge efficacy, and lightning conductor installation, were recorded using measuring instruments as detailed below.

1. Flammable gases were measured as a %LEL‐UEL of flammable gas using a flammable gas detector in the working area, both when refuelling and in the normal working atmosphere.
2. The heat ignition source measurement was performed by a thermography inspector, who took thermal images of surface materials, such as vehicle exhausts, engine bonnets, dispenser cabinets, cash registers, plugs, fans, computers, and cellphones in the gasoline stations working areas. The 95th percentile of the temperature was obtained using the IRSoft program to analyze, process, and archive the images recorded by the thermal imager used in the testing and programmed by the thermography inspector.
3. Electrostatic discharge efficacy was measured using electrostatic field meters on the surfaces of materials in the working area, such as dispenser cabinets, cellphones, the bodies of vehicles, fuel containers, and the worker's bodies.
4. The comprehensive installation of lightning conductors in the gasoline station areas was checked using the gasoline station questionnaires and observations.

### 2.3 Fire hazardous zone classification at gasoline stations

A fire hazardous zone (FHZ) is defined according to IEC 60079‐10‐1:2008‐Explosive atmospheres‐Part 10‐1: Classification of areas‐explosive gas atmospheres^15^; and the Ministry of Energy (Thailand) regulations (regarding electrical installation and lightning protection installation at gasoline stations^13^). Any FHZ can be classified as one of two zones (fire hazardous zone‐I (FHZ‐I) and fire hazardous zone‐II (FHZ‐II)) according to the definitions below.

Fire hazardous zone‐I (FHZ‐I) is an area in which there is a mixture of gases or vapors in the atmosphere with appropriate risk of fire ignition during normal operations, maintenance, or flammable leakage. There is high probability that a 100% LEL for flammable gases will be present between 10 and 1000 hours per year in normal conditions, such as within a 1.5 meter radius of the dispenser installation point and refuelling area.

Fire hazardous zone‐II (FHZ‐II) is an area in which there is a mixture of gases or vapors in the atmosphere with appropriate risk of fire ignition. While vapor or mist is not likely to occur in normal operations, if it does occur, it will persist for a short period. There is a high probability that a 100% LEL for flammable gases will be present for less than 10 hours per year within a 1.6 to 5 meter radius of the dispenser installation point.

### 2.4 Fire risk assessment on hazardous zones at gasoline stations

Two hazardous zones around the dispensers, which were used while refuelling at gasoline stations, were considered in the determination of fire risk. They are detailed as follows.

#### 2.4.1 The fire risk assessment on fire hazardous zone I (FHZ‐I)

The fire risk assessment on FHZ‐I was performed by analyzing the applied matrix of multipliers of opportunity and the subsequent severity of the resulting fire, which was a risk assessment application from OHSAS18001:2007^16^ and Australian/New Zealand (AS/NZS) 4360:2004^17^ Risk Management Standard, as per Equation (1) and the applied matrix in Table 1.

(1) FRi=S×OP

where FRi is the fire risk assessment on fire hazardous zone I (fire risk groups are ranked 1‐4), S is severity of an incident at the gasoline station (four levels in this study), OP is opportunity of occurrence of the hazard in FHZ‐I (ranked at levels of 1‐3).

**TABLE 1:** Fire risk assessment matrix multipliers for fire hazardous zone‐I and zone‐II (FHZ‐I and FHZ‐II)

Opportunity of hazard occurrence (FHZ‐I)	Likelihood of hazard occurrence (FHZ‐II)	Severity of incident to the gasoline station (FHZ‐I and II)			
		Slightly harmful (1)	Harmful (2)	Extremely harmful (3)	Catastrophic (4)
Likely (3) (OP: score of ≥3)	Likely (3) (LO: ≥75%)	Trivial risk (1 = score of 1‐3)	Moderate (2 = score of 4‐6)	Substantial risk (3 = score of 7‐9)	Intolerable risk (4 = score of 10‐12)
Unlikely (2) (OP: score of 2)	Unlikely (2) (LO: ≥50%‐74%)	Trivial risk (1 = score of 1‐3)	Moderate (2 = score of 4‐6)	Moderate (2 = score of4‐6)	Substantial risk (3 = score of 7‐9)
Highly unlikely (1) (OP: score of 1)	Highly unlikely (1) (LO: <50%)	Trivial risk (1 = score of 1‐3)	Trivial risk (1 = score of 1‐3)	Trivial risk (1 = score of 1‐3)	Moderate (2 = score of 4‐6)

Applied from OHSAS18001:2007^14^, ^16^.

The fire risk of FHZ‐I (FRi) was determined by analyzing the results in the matrix of multipliers of opportunity levels and the severity ranking levels, and then classifying the four fire risk groups; intolerable risk (4 = score of 10‐12); substantial risk (3 = score of 7‐9); moderate risk (2 = score of 4‐6); and trivial risk (1 = score of 1‐3), as shown in Table 2.

**TABLE 2:** Fire risk (FRi or FRii) assessment rating

Rating	Score	Description	Suggested mitigating activity
4	10‐12	Intolerable Risk	The following activities should be performed:
			‐ Engineering improvement: vapor recovery system (VRS) installation; regular checking of electrostatic discharges; grounding installation (for refuelling into containers); and fire hazardous zone designating and signposting.
			‐ Strictly controlled management of fire ignition sources.
3	7‐9	Substantial Risk	The following activities should be performed:
			‐ Engineering improvement: regular checking of electrostatic discharges; grounding installation (for refuelling into containers); and fire hazardous zone designating and signposting.
			‐ Strictly controlled management of fire ignition sources.
2	4‐6	Moderate	Fire hazardous zone designating and signposting should be performed and there should be strictly controlled management of fire ignition sources.
1	1‐3	Trivial Risk	There should be strictly controlled management of fire ignition sources.

Applied from AS/NZS 4360:2004 Risk Management Standard^15^, ^17^.

The severity (S) of an incident at the gasoline station was determined by the risk level classification from the severity level, in which levels of severity were divided into four levels: 4 = catastrophic; 3 = extremely harmful; 3 = harmful; and 1 = slightly harmful. The severity was indicated as the highest level (level 4) for all cases because fire damage would be severe and catastrophic.

The opportunity of hazard occurrence in FHZ‐I was divided into three levels according to the level of aggregate results of fire possibility in FHZ‐I. The ranking was as follows: 3 = likely; ≥3, 2 = unlikely; and 1 = highly unlikely. The following criteria were used to estimate the aggregate results of fire possibility in FHZ‐I: fire ignition source observation (0 = no, 1 = yes) (sources included cellphone use, defective or non‐standard electrical apparatuses, not turning off the engine, and smoking), plus the scores from factors causing the fire: 1 = a 1.3%‐7.4% flammable gas LEL range (gasoline = 1.4%‐7.4%LEL‐UEL, diesel = 1.3%‐6.0%LEL‐UEL); 1 = surface temperature of materials higher than 85°C; and 1 = static electricity measuring more than 5 ohm.

#### 2.4.2 Fire risk assessment of fire hazardous zone‐II (FHZ‐II)

The fire risk assessment on FHZ‐II was performed by analyzing the results of the matrix of multipliers of likelihood and the subsequent severity of the resulting fire, which applied the risk assessment from OHSAS18001,^16^ as per Equation (2) and the matrix, as follows:

(2) FRii=S×LO

where FRii is the fire risk assessment in fire hazardous zone II, S is severity of the incident at a gasoline station, and LO is the likelihood of occurrence of the hazard at the gasoline station.

The fire risk of FHZ‐II (FRii) and the severity (S) of the incident were determined as in the first equation presented in the matrix of Tables 1 and 2.

The likelihood of occurrence (LO) of the hazard at the station was estimated from the result of Equation (3), as follows:

(3) LO=(SL/∑SL)×100

where LO is the likelihood of occurrence of the hazard, SL is the result of the score of likelihood in related factors, and ∑SL is the summary of the score of likelihood in related factors (score out of 30).

LO was divided into three levels according to percentage criteria with regard to the level of occurrence likelihood (ranking of occurrence likelihood: 3 = highly likely [≥75%]; 2 = likely [≥50% ‐ <75%]; and 1 = unlikely [<50%]).

The criteria used to assess the likelihood of danger from correlated factors that could cause a fire consisted of 10 factors (L1‐L10: L1 = workers; L2 = working hours; L3 = fire incident frequency; L4=%LEL‐UEL of flammable gas; L5 = heat ignition source; L6 = frequency of electronic device use; L7 = electrostatic discharge on materials around the working area; L8 = electrostatic current on the gasoline station worker's body; L9 = installation of electrostatic discharge control; and L10 = lightning conductor installation. These factors were derived from the questionnaire and observations at gasoline stations. The risk behavior of workers at each station regarding L3, L6, L9, and L10 was analyzed on the basis of the questionnaire and observations. Data on L4 were collected using flammable gas detectors, data on L5 were collected by the thermography inspector, and L7 and L8 were measured using an electrostatic field meter. These factors, derived from the fire triangle theory, are detailed in Table 3.

**TABLE 3:** Fire risk factors as a likelihood of occurrence (LO) in FHZ‐II and their respective scores (n = 47 stations)

Fire risk factors (summation score; ∑SL = 30)	n (%)
Number of workers per station (persons) (L1)	
1‐3 (score of 1)	2 (4.26)
4‐6 (score of 2)	9 (19.15)
More than 7 (score of 3)	36 (76.60)
Working hours and frequency (h/day or h/year) (L2)	
<4 h/day or <1300 h/year (score of 1)	0 (0)
4‐8 h/day or 1300‐2500 h/year (score of 2)	0 (0)
>8 h/day or >2500 h/year (score of 3)	47 (0)
Sparking and fire frequency (L3)	
<1 time every 5 years (score of 1)	26 (55.32)
1 time per year (score of 2)	5 (10.64)
>1 time per year (score of 3)	16 (34.04)
Range of % LEL‐UEL of flammable gas (1.3%‐7.4%) (L4)	
Level 1: not in the range of % LEL‐UEL (score of 1)	0 (0)
Level 2: in the range of % LEL‐UEL (score of 3)	47 (100)
Mean (SD)	6.19 (5.17)
Median (Min: Max)	5 (2:26)
95th percentile of temperatures of heat ignition sources (°Celsius) (L5)	
<38 (score of 1)	0 (0)
≥38 (score of 3)	47 (100)
Frequency of using and carrying electronic devices by workers (h) (L6)	
<1 h/day using and carrying (score of 1)	0 (0)
1‐4 h/day using and carrying (score of 2)	0 (0)
>4 h/day using and carrying (score of 3)	47 (0)
Maximum level of electrostatic discharge on materials (ohm) (L7)	
<5 ohm (score of 1)	47 (0)
≥5 ohm (score of 3)	0 (0)
Maximum level of electrostatic discharge on workers’ bodies (ohm) (L8)	
<5 ohm (score of 1)	47 (0)
≥5 ohm (score of 3)	0 (0)
Electrostatic discharge installation (L9)	
Installed in more than 85% of an area (score of 1)	2 (4.26)
Installed in 50%‐85% of an area (score of 2)	22 (46.80)
Installed in <50% of area (score of 3)	23 (48.94)
Lightning conductor installation (L10)	
Installed and routinely maintained (score of 1)	10 (21.28)
Installed but not maintained (score of 2)	12 (25.53)
Not installed (score of 3)	25 (53.19)

### 2.5 Statistical analysis

The data were analyzed using STATA version 10 software, and descriptive statistics were used to define fire risk and classify hazardous zones. The Kruskal‐Wallis test and a chi‐squared test were used for correlation analysis of factors and fire risk. A 95% confidence interval (CI) was calculated and the statistical significance was identified at a *P*‐value < .05.

## 3 RESULTS

### 3.1 Gasoline station characteristics

Of the 47 representative stations, 14.89% were located in urban areas, 61.70% in suburban areas, and 23.40% in rural areas. In total, 27 of the gasoline stations (57.45%) were open for 16 hours a day (06.00‐22.00 hours) and 20 stations (42.55%) were open 24 hours a day. The average number of fuel dispensers was 23 ± 12 nozzles (min:max = 8:48) and none had a vapor recovery system (VRS) installed. The daily gasoline sold averaged 3382.77 ± 2382.95 L (min: max = 600:13 400). The average concentration of VOCs was 410.0 ± 172.0 ppm (minimum: maximum = 158:810) and it was shown that 30 stations (63.82%) had more than 300 ppm of total VOCs.

The gasoline stations were classified according to service type characteristics, where 14 stations (29.79%) were type IV (fuel dispensers, oil storage tanks, office, maintenance store, mini‐mart, coffee shop, food court), 21 stations (44.68%) were type III (fuel dispenser house, oil storage tanks, office, maintenance store, mini‐mart, coffee shop), six stations (12.77%) were type II (fuel dispensers, oil storage tanks, office, maintenance store), and six stations (12.77%) were type I (fuel dispensers, oil storage tanks, office).

### 3.2 Characteristics of the fire risk factors considering the likelihood of occurrence

The criteria for factors concerning the likelihood of occurrence consisted of 10 factors of fire risk and they are presented in Table 3. Workers of all the stations (n = 47) had used a cellphone more than 4 hours a day (out of more than eight working hours) at all stations, and flammable gas was in the range 1.3%‐7.4%LEL‐UEL with an average of 6.19 ± 5.17%. A total of 25 gasoline stations (53.19%) did not have a lightning conductor installed, whereas 25.53% of them had no lightning conductor inspections. All the gasoline stations were equipped with electrostatic discharge devices, but the installations did not cover more than 85% of an area (95.75%) and 21.28% had no inspections of electrostatic discharge apparatuses. From the observation data, it was found that most of the stations had a working area within a 1.5 meter radius of the dispenser, and all 47 stations had fire ignition sources around the dispenser (FHZ‐I) from the using and carrying of electronic devices by workers for more than 4 hours a day. The 95th percentile of temperature of heat ignition sources of 38°C or higher was monitored at all stations and 16 stations (34.04%) experienced electrical short incidents on the apparatuses in the gasoline station area.

### 3.3 Fire hazardous zone and fire risk assessment

#### 3.3.1 Fire risk assessment of fire hazardous zone I (FHZ‐I)

The opportunity of occurrence of hazard (LO) score was not higher than 3. The result of the FRA on FHZ‐I showed that 40 stations had an intolerable fire risk and seven stations were at a substantial risk.

#### 3.3.2 Fire risk assessment of fire hazardous zone II (FHZ‐II)

From the results of the fire risk assessment on FHZ‐II, it was estimated that 10, 18, and 19 stations had intolerable, substantial, and moderate fire risks, respectively (Table 4).

**TABLE 4:** Fire risk assessment in fire hazardous zones at the dispenser areas (n = 47)

Fire risk level	Fire risk ranking	
	FHZ‐I; n (%)	FHZ‐II; n (%)
1: Trivial risk	0 (0)	0 (0)
2: Moderate risk	0 (0)	19 (40.43)
3: Substantial risk	7 (14.89)	18 (38.30)
4: Intolerable risk	40 (85.11)	10 (21.28)

### 3.4 Correlation between fire risk in hazardous zones and number of dispensers, location of the station, gasoline sold, and service type

There was a significant correlation between the gasoline station location and the intolerable risk level. Intolerable fire risks in FHZ‐II were mostly found in urban areas (57.15%), whereas all of the stations in urban areas had an intolerable level of risk in FHZ‐I. Higher amounts of gasoline sold daily significantly increased the fire risk in FHZ‐II. Data showed that stations selling over 1500 L of gasoline resulted in an intolerable risk at most stations in FHZ‐I (32 out of 40 stations).

Intolerable fire risk in FHZ‐I was noted at 28 gasoline stations (65.50%) of service type IV and III. All service type I and type II stations (12 stations (35.50%) which had no maintenance service or food court) had intolerable risks in FHZ‐I. The level of risk was increased in a FHZ‐I where there were more than 32 nozzles. A concentration of VOCs higher than 300 ppm was not significantly correlated with intolerable fire risk (Table 5).

**TABLE 5:** Correlation between fire risk level in hazardous zones and the location of the station, service type, number of dispensers, gasoline sold, and concentration of VOCs (n = 47)

Factors	FRA‐FHZ‐I n (%)			FRA‐FHZ‐II n (%)				
	Substantial	Intolerable	*P*‐value	Moderate	Substantial	Intolerable		*P*‐value
Location of the station			.486				.020*	
Urban (n = 7)	0 (0)	7 (100)		1(14.3)	2 (28.6)	4 (57.2)		
Suburban (n = 29)	5 (17.2)	24 (82.7)		10 (34.5)	13 (44.8)	6 (20.7)		
Rural (n = 11)	2 (18.2)	9 (81.8)		8 (72.7)	3 (27.3)	0 (0)		
Gasoline sold (liters/day)			.799				.041*	
<1500 (n = 9)	1(11.1)	8 (88.9)		6 (66.7)	1(11.1)	2 (22.2)		
1500‐3199 (n = 14)	3 (21.4)	11 (78.6)		3 (21.4)	7 (50.0)	4 (28.6)		
3200‐4849 (n = 12)	2 (16.7)	10 (83.3)		4 (33.3)	8 (66.7)	0 (0)		
≥4850 (n = 12)	1(8.3)	11 (91.7)		6 (50.0)	2 (16.7)	4 (33.3)		
Service type of station			.367				.386	
Type IV (n = 13)	2 (15.4)	11 (84.6)		6 (46.2)	4 (30.7)	3 (23.1)		
Type III (n = 22)	5 (22.7)	17 (77.3)		8 (36.4)	10 (45.5)	4 (18.2)		
Type II (n = 6)	0 (0)	6 (100)		1(16.7)	2 (33.3)	3 (50.0)		
Type I (n = 6)	0 (0)	6 (100)		4 (66.7)	2 (33.3)	0 (0)		
Number of dispensers			.808				.656	
<12 (n = 8)	1(12.5)	7 (87.5)		5 (62.5)	1(12.5)	2 (25.0)		
12‐17 (n = 11)	2 (18.2)	9 (81.8)		5 (45.5)	4 (36.4)	2 (18.2)		
18‐31 (n = 15)	3 (20.0)	12 (80.0)		4 (26.7)	7 (46.7)	4 (26.7)		
>32 (n = 13)	1(7.7)	12 (92.3)		5 (38.5)	6 (46.2)	2 (15.4)		
Concentration of VOCs			.650				.091	
<300 ppm (n = 17)	2 (11.7)	15 (88.3)		4 (23.5)	10 (58.8)	3 (17.6)		
≥300 ppm (n = 30)	5 (16.7)	25 (83.3)		15 (50.0)	8 (26.7)	7 (23.3)		

* Significant at *P*‐value < .05.

## 4 DISCUSSION

The fire risk analysis in this study showed that there was a greater frequency of intolerable fire risk in FHZ‐I compared to FHZ‐II. The least frequently observed risk level was the moderate risk level, which only tended to be observed in FHZ‐II. The reason for the intolerable risk indicated in FHZ‐I could be that all 47 gasoline stations in this study had concentrations of flammable gas in a range where it could ignite and explode in addition to the risks of having a fire ignition source and additive heat ignition. The previous studies’ findings showed that concentrations of flammable gas can be reduced by VRS installation at fuel dispensers, which can reduce fuel vapor by 80.0%‐99.9%.^18^, ^19^ In Thailand, the Ministry of Energy have promoted the installation of VRS at gasoline stations under their control in Bangkok, the capital city, and its perimeter areas since 2007.^13^ Our study indicated that VRS installation was unavailable in the city of Khon Kaen's gasoline stations, which should be considered for fire protection. The primary fire ignition source was from the workers’ behavior in using and carrying electronic devices in the fire hazardous zone (within 1.5 meters of the dispenser area). In parallel with regularly documented inspections for electrostatic discharges and 100% installation of grounding for refuelling into containers, signposting of fire hazardous zones with prohibition of mobile phone use should be strictly enforced to free such zones of fire ignition sources.

A significant correlation was found between fire risk levels in FHZ‐II and a station's location. That is consistent with the previous report of higher health risk among workers at urban and suburban gasoline stations compared to rural stations.^20^ Gasoline stations in suburban zones were located along the main highway connecting Bangkok, the capital city to the countries of the Mekong sub‐region, relating to our previous study which showed that a high benzene concentration in the air correlated with the daily amount of gasoline sold.^21^ This study supports the theory that the amount of gasoline sold per day was significantly correlated with fire risk in FHZ‐II. This might be explained by the fact that increased levels of service caused more emissions of flammable gas. The likelihood of occurrence from fire ignition sources showed that all gasoline workers had risk behaviors in their use of cellphones or electronic devices for more than 4 hours a day. Those high risks must be controlled by eliminating the unsafe working behavior and reducing the unsafe conditions by performing regular inspections and ensuring installation of electrostatic discharge equipment and lightning conductors in hazardous zones.

Besides, other factors that may cause fire risks at gasoline stations included characteristics of service type and the number of dispensers. The FRA assessment showed an intolerable fire risk at most stations (59.57%) of service type IV and III. Those types of station contain facilities which accommodate many people simultaneously, including mini‐marts, food courts, and cafes, and most of these stations had more than 18 fuel dispensers, which may release flammable gases with higher levels of %LEL and emission of VOCs. Certain aspects of those gasoline stations led to increased fire risk factors; for example, the number of gasoline station workers who often exhibited many risk behaviors, such as usage of cellphones, working longer than 8 hours per day, and running small shops, some of which had fires and sparking. Therefore, the establishment of strict control by safety management and the use of the fire protection method, as previously mentioned, must be enforced.

## 5 CONCLUSIONS

This study of fire risk assessment in hazardous zones of gasoline stations showed that for fire hazardous zone‐I (within a 1.5 meter radius of the dispenser), 85.11% of the stations surveyed had an intolerable level of risk. Meanwhile, for fire hazardous zone‐II (within a 1.6 to 5 meter radius of the dispenser), 21.28% of the stations surveyed had an intolerable level of risk and 38.30% had a substantial risk. The factors resulting in high fire risk were flammable gas in the range 1.3%‐7.4% LEL‐UEL and worker behaviors within a 1.5 meter radius of the dispenser (FHZ‐I), especially with regard to the fire ignition risk from cellphone use and heat ignition sources inside FHZ‐II zones. The gasoline station locations and the quantity of fuel sold correlated with the fire risk of FHZ‐II at gasoline stations in Khon Kaen, Thailand. No VRS system installation was found at fuel dispensers, and there was an average of 23 ± 12 dispensers per station, in this study.

## DISCLOSURE

*Ethical approval:* The study obtained ethical approval from Khon Kaen University ethics committee, Thailand, No. HE612102. *Informed Consent:* All participants gave informed consent prior to taking part in the study. *Registry and the Registration No. of the Study/Trial:* N/A, *Animal Studies* ***:*** N/A, *Conflict of Interest:* Authors declare no conflict of interests in writing this article.

## AUTHOR CONTRIBUTIONS

Sunisa Chaiklieng invented and designed the study, and was the principal investigator and manuscript writer; Thawatchai Dacherngkhao participated in acquisition of data; Pornnapa Suggaravetsiri performed the data analysis and wrote the discussion; and Vichai Pruktharathikul had participated in the discussion.
